# Multimodal theories of recognition and their relation to Molyneux's question

**DOI:** 10.3389/fpsyg.2014.01547

**Published:** 2015-01-08

**Authors:** Nicholas Altieri

**Affiliations:** ISU Multimodal Language Processing Lab, Department of Communication Sciences and Disorders, Idaho State UniversityPocatello, ID, USA

**Keywords:** Molyneux, multisensory perception, nativism debate, empiricism, associative learning

In the seventeenth century, William Molyneux posed his now famous question to John Locke, a question about the contents of human sensory experience, the relation between cross-modal perceptions, and the origins of sensory content more generally. Locke (as cited by Molyneux's Question, n.d.^1^) paraphrased Molyneux's Question as follows:

“Suppose a man born blind, and now adult, and taught by his touch to distinguish between a Cube, and a Sphere of the same metal, and nighly of the same bigness, so as to tell, when he felt one and t'other, which is the Cube, which the Sphere. Suppose then the Cube and Sphere placed on a Table, and the Blind Man to be made to see. Quære, whether by his sight, before he touched them, he could now distinguish, and tell, which is the Globe, which the Cube (Locke, 1694/1979).”

Since then, empiricists and nativists have argued about these issues. Empiricists, who take all sensory content to be derived from experience, have traditionally argued for the negative conclusion—a blind man would not differentiate the cube from the sphere on first sight. Nativists, who believe that we are born with at least some core conceptual resources, have often championed the positive view: A transfer of content from haptics to vision would occur, thus enabling the blind man to differentiate between the two shapes. Today, a burgeoning interest in multimodal processing has again resurrected Molyneux's Question. Sometimes authors have identified and addressed this set of problems by name (Held et al., 2011; Connolly, 2013, 2014). Other researchers raise very similar questions but pose them in a modern guise. They ask: How we should interpret data from multimodal experiments in the behavioral and neural sciences (e.g., Fowler, 2004; Wallace et al., 2006; Connolly, 2014)?

I show why the traditional answers, “yes” by Nativist and “no” by the Empiricist, are too simple^2^. Specifically, I begin by arguing that at least two nativist theories can predict a “no” answer to Molyneux's Question. I then go on to argue that theories promoting a common coding scheme across modalities are the nativist accounts that predict an affirmative answer. This is important as many influential accounts of multisensory perception in the field of speech recognition and elsewhere shares these features and should therefore be explored in light of these issues. A framework in the field of Psychology known as *direct perception*, for instance, argues that speech and other phenomena are recognized “directly” as dynamic-gestural events. This is true regardless of whether cues are obtained via the auditory, visual, or even tactile modalities (Rosenblum et al., 1997; Fowler, 2004). Crucially, the information accessed by the perceiver is *amodal* and hence not tied to a specific modality, such as the auditory. I also illustrate how these issues make predictions for theories of speech perception and our interpretation of phenomena such as the McGurk effect (McGurk and MacDonald, 1976). Finally, I describe how ongoing developments in behavioral and reaction-time methods can be used to distinguish the different nativist and empiricist accounts of perception discussed in this paper.

## Nativist theories and molyneux's question

In this section, I show how at least two nativist theories predict a negative answer to Molyneux's Question; I refer to these as the *Common Geometry* and *Domain specific modular theory*^3^. I then highlight how accounts associated with *direct perception* predict an affirmative response.

1. Common Geometry Theory
  Consider the following example: suppose an individual has an innate concept of a three-dimensional round object labeled *sphere*. We know that mathematically, *sphere* may be defined as a set of points in space that are equal-distant from an arbitrary point. Notice that *sphere* is an abstract concept that is not bound to any particular sensory modality, although it can be mapped to visual, tactile, and perhaps even an auditory representation given sufficient perceptual experience. The crucial feature that distinguishes this theory from most empiricist frameworks is that the innate concept guides perception in such a way that experience gradually allows one to map sensations to an inborn primitive or idea, and not the other way around. This may perhaps best be illustrated in the following way. Most individuals have an intuitive notion of the concept *sphere*; however, will they be able to distinguish a sphere from another object using auditory cues, such as through reverberation and shadow effects, without sufficient experience? The answer is probably “no.” This is not because one lacks fundamental knowledge of “sphereness,” but rather, because that knowledge is inaccessible in a specific modality without sufficient practice (even though this hypothetical individual was born with normal hearing!).
  It is therefore straightforward to see why this version of nativism predicts a negative answer to Molyneux's Question. On one hand, abstract knowledge of specific events or objects might be present prior to experience, be they spoken language or spheres. However, that knowledge can only be associated by a specific sensory modality through experience.
2. Domain Specific Modular Theory
  One might easily propose an amended version to the theory above that also fails predict an affirmative response. Suppose that besides the innateness of concepts, we make the additional assumption that certain phenomena, such as spoken language, are innately bound to or otherwise associated with a specific modality through some sort of module. According to this position, language and phoneme perception might be thought of a modular auditory function (e.g., Chomsky and Halle, 1968) while other functions could be subsumed by tactile or visual-based modules (e.g., letter perception) that engage automatically when provided with domain specific inputs (Fodor, 1983).
  As it currently stands, this nativist theory also predicts a “no” answer to Molyneux's Question. That is, unless we make at least one further assumption of an automatic mechanism that translates the information into a common code subsequent to auditory (or visual) perceptual coding. To see exactly why, consider the case of the McGurk effect in which an auditory /ba/ is dubbed over a visually articulated “ga” (McGurk and MacDonald, 1976). The visual “ga” causes the auditory phoneme /b/ to be assimilated to a phonemic category that shares a similar manner of articulation as /b/, while sharing a similar place of articulation as the viseme “ga.” The perception is typically “da” or “tha” in normal-hearing adults. The important thing is that the association of auditory information to lip movements is not innate since the visual perceptual mechanisms do not have immediate access to auditory modular outputs. The prediction is that someone born blind and suddenly has their sight restored will hear “ba” regardless of the lip-movements it accompanies. One may use analogous reasoning to understand why a congenitally blind individual with restored vision should be able to identify an object, say a sphere, through the tactile modality while remaining unable to automatically associate it with visual information.
3. Common Code Theory
  I argue that in order for Molyneux's Question to be answered with a “yes,” one further assumption must be made. An implicit yet fundamental assumption must be that multisensory perception involves either the innate representation of objects across modalities, or perhaps the automatic translation of object representations into a common code across modalities. Thus, recognition must occur through or representations must be translated into a common amodal code (e.g., dynamic events or gestures). This appears to be exactly what the “intermodal feature binding theory” reviewed by Connolly (2014) as well as Gibsonian theories and related theories of direct perception typically assume. In speech perception, the dynamic event is perceived, such as the gesture producing “ba” or “ga,” rather than an auditory code or discretized phoneme. As such, /ba/ and visual “ga” are encoded as gestural events regardless of the sensory modality through which the information is obtained. The association between auditory and visual speech events naturally occurs through an automatic binding process since the perceptual event exists in a common code across modalities in the first place. Per Molyneux's Question, a person born blind with sight suddenly restored should have the capability of identifying a sphere through vision alone. This is because the tactile code and information accrued through vision subside in an amodal format.

## Predictions

Empirically testing Molyneux's Question is not straightforward. A recent examination, for instance, was carried out using congenitally blind participants who had their sight restored and testing them on their ability to match sight to touch (Held et al., 2011). Participants failed to match 3D objects to tactile representations prompting the authors to suspect a probable “no” answer to Molyneux's Question. One potential shortcoming of Held et al.'s (2011) study was that the newly sighted may have poor visual representations of depth cues (cf. Connolly, 2013; Schwenkler, 2013). This led Connolly (2013) and Schwenkler (2013) to discuss the possibility of re-running the experiment using raised line-drawings rather than 3D objects. I agree that this is a viable improvement, although argue below that other predictions for the newly sighted can likewise get to the theoretical core of Molyneux's Question.

**Figure 1:**
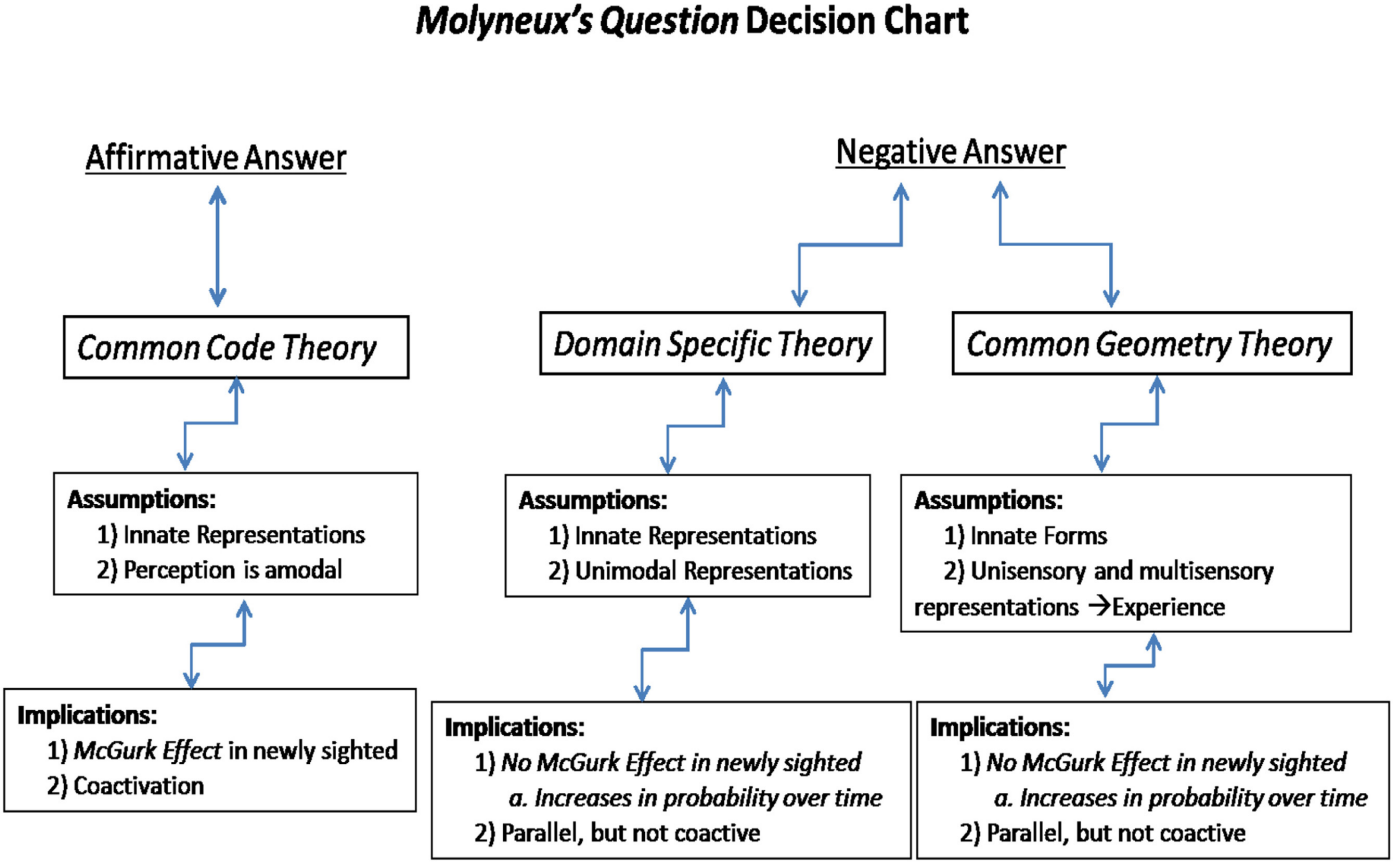
**Predictions and features of the three nativist frameworks of multimodal perception**.

Figure 1 shows a diagram of predictions for categorizing the three nativist theories of perception. First, if the perceptual primitives of auditory, visual, and tactile sensory encoding are coded as amodal gestures, as in *Common Code Theory*, then encoding of incoming information should be fast, efficient, and form holistic units. This fast and efficient processing strategy is sometimes referred to as “coactivation.” It can be assessed using speeded recognition detection paradigms that manipulate perceptual workload by varying the number of modalities present in a display (cf. Altieri and Townsend, 2011, for an example in audiovisual integration). An additional prediction is an “innate” ability to associate cross-modal events—for example, the prediction that the newly sighted, or infants should perceive the McGurk effect (Rosenblum et al., 1997).

The two nativist theories predicting a negative response diverge in their predictions regarding the McGurk effect and multisensory processing strategy (coactive, vs. parallel or serial). The difference in prediction lies in the fact that the first theory allows for an innate association mechanism across modalities. While theory 2, for example, allows for a universal grammar on phonemic perception assessable through the auditory modality (or visual in congenitally deaf individuals), the association between modalities must still be learned. Therefore, the McGurk effect should not be present in the newly sighted but become observed with an increasingly higher probability over time. Information must be processed separately in parallel auditory and visual processing channels, although cross-modal connections strengthen through learning. These theories also predict parallel rather than coactive processing; an observation reported in neurocognitive multimodal speech studies (Altieri and Wenger, 2013).

In conclusion, this article discussed some limitations in differentiating learned association theories from nativist ones from the perspective of Molyneux's Question. This paper did not aim to promote one framework over another, although it did illustrate how Molyneux's Question can prove beneficial for sorting out questions concerning finer grained theories of intermodal binding. Clearer discussion on theories of multimodal binding is especially important in light of continued advances in perceptual sciences alongside neuro-imaging technology.

### Conflict of interest statement

The authors declare that the research was conducted in the absence of any commercial or financial relationships that could be construed as a potential conflict of interest.

## Footnotes

^1^In the Internet Encyclopedia of Philosophy. Retrieved from http://www.iep.utm.edu/molyneux/.

^2^A variety of empiricist (perceptual learning and/or segregation of the senses) and nativist accounts (inborn concepts, common geometrical representations; Van Cleve, 2007) have been proposed for negative and affirmative answers respectively (cf. Molyneux's Question, n.d.).

^3^Experimentally differentiating theories 1 and 2 is complex and beyond the scope of this report.
